# A Model for Damage Load and Its Implications for the Evolution of Bacterial Aging

**DOI:** 10.1371/journal.pgen.1001076

**Published:** 2010-08-26

**Authors:** Lin Chao

**Affiliations:** Section of Ecology, Behavior, and Evolution, Division of Biological Sciences, University of California San Diego, La Jolla, California, United States of America; University of Toronto, Canada

## Abstract

Deleterious mutations appearing in a population increase in frequency until stopped by natural selection. The ensuing equilibrium creates a stable frequency of deleterious mutations or the mutational load. Here I develop the comparable concept of a damage load, which is caused by harmful non-heritable changes to the phenotype. A damage load also ensues when the increase of damage is opposed by selection. The presence of a damage load favors the evolution of asymmetrical transmission of damage by a mother to her daughters. The asymmetry is beneficial because it increases fitness variance, but it also leads to aging or senescence. A mathematical model based on microbes reveals that a cell lineage dividing symmetrically is immortal if lifetime damage rates do not exceed a threshold. The evolution of asymmetry allows the lineage to persist above the threshold, but the lineage becomes mortal. In microbes with low genomic mutation rates, it is likely that the damage load is much greater than the mutational load. In metazoans with higher genomic mutation rates, the damage and the mutational load could be of the same magnitude. A fit of the model to experimental data shows that *Escherichia coli* cells experience a damage rate that is below the threshold and are immortal under the conditions examined. The model estimates the asymmetry level of *E. coli* to be low but sufficient for persisting at higher damage rates. The model also predicts that increasing asymmetry results in diminishing fitness returns, which may explain why the bacterium has not evolved higher asymmetry.

## Author Summary

Almost all living organisms deteriorate with time through the process of aging or senescence. Because most studies on senescence examined organisms possessing a juvenile state, it was thought that bacteria, which reproduce by producing two apparently identical daughter cells, were immortal and not senescent. Recent studies have demonstrated that bacteria senesce because one daughter is allocated a larger share of the mother's load of non-genetic damage. Nonetheless, it is still equivocal whether bacterial senescence renders them mortal. I have developed a model that demonstrates that bacteria can be immortal if they experience damage below a threshold rate. A fit of the model to data shows that bacteria grown under standard laboratory conditions are immortal because they encounter a rate below the threshold. Because bacteria often experience higher damage rates in nature, it is likely that bacteria are generally mortal. The allocation of more damage to one daughter and the resulting mortality is the price bacteria pay to survive higher damage rates. These results suggest that senescence originated with the evolution of the first single-celled organisms and that it is ancestral in all multicellular organisms.

## Introduction

Evolution by natural selection generally produces phenotypes that maximize fitness, but many factors can interfere. Genetic constraints can lead to a suboptimal phenotype, but an optimal phenotype may not be achieved even when an optimal genotype is possible. If the rate of deleterious mutations is sufficiently high, selection is unable to eliminate all mutations and a mutation-selection equilibrium at a lower fitness ensues. An asexual population has at equilibrium a mean fitness of
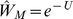
(1)where *U* is the deleterious genomic mutation rate [1], [2]. The mutational load equals 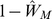 (references [3]–[5]).

However, an optimal phenotype can also be prevented by the direct action of non-heritable damage. Bones can be broken, muscles torn, and macromolecules oxidized. All these lower fitness despite the perfection of the genotype. Although the study of deleterious mutations is long standing in evolution [1]–[7], interest in damage is recent. The transmission of deleterious mutations across generations follows the rules of genetics. While damage is not heritable, it can still be transmitted from mother to daughter and its transmission rules are just being explored as an evolutionary phenomenon [8]–[11]. Here I develop the concept of a damage load and analyze its evolutionary consequences. Mutational and damage loads may appear on the surface similar, but key and fundamental differences are revealed by a comparison. Because recent experimental work has stimulated an interest in the effects of damage in microbes [12]–[14], the analysis focuses on a single-celled organism reproducing by binary fission.

A model for damage load can be developed by allowing the generation of damage, the operation of selection, and the attainment of the ensuing damage-selection equilibrium. Recent models have in fact used such an approach to examine the evolution of transmission rules for damage, i.e. how a mother cell distributes her damage to her two daughter cells [8]–[11]. However, with the exception of the most recent model by Erjavec *et al.* [11] all of these models were limited because a key difference between damage and mutations is the timing of their effects. In metazoans, the consequences of damage are immediate because the soma is affected. Mutational damage to the germline is delayed to the next generation. Because somatic mutations are not inherited through the germline, they are effectively non-heritable in most organisms and equivalent to damage for the present analysis. For single-celled organisms damage such as oxidized proteins has immediate effects, while genetic damage is again delayed in expression. If damage acts immediately, an early event during the lifetime of an organism has more impact than a later one. More importantly, an early damage can extend generation time and expose the organism to even more damage.

To incorporate the timing of damage, a new model was developed. The model shared some similarities with the one by Erjavec *et al.*, but it assumed instead that the effect of damage was linear. Ackermann *et al.* [8] examined a range of damage effects, including a linear relationship, but their effects were mapped directly to fitness and thus did not incorporate the timing of damage. Given that evidence supporting either a linear or higher order effect of damage is lacking, a linear assumption is parsimoniously reasonable and provides more statistical power by reducing the number of parameters. Linearity additionally allows for simpler but explicit solutions and facilitates fitting data to estimate key parameters in the model.

## Model

Let *k*_0_ be the amount of damage a mother cell receives at birth. She immediately acquires new damage and, if λ is the intrinsic rate of damage, her damage at any time *t* is
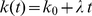



To divide into two daughter cells, the mother cell is assumed to build up an intracellular product *P* to a checkpoint Π. Assuming that damage hinders function linearly, *P* accumulates at a rate
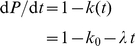
(2a)

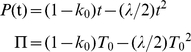
(2b)by integration and letting *t* = *T*_0_ and *P*(*T*_0_) = Π when the mother cell divides and *T*_0_ is her doubling time. The integration constant *P*(0) is set to zero because a new pool of the product *P* is assumed to be built *de novo* for every cell division. If *k*_0_ = λ = 0, *T*_0_ = Π in Equation 2b. Thus, Π represents both the checkpoint and the shortest possible doubling time achieved by the fittest and damage-free bacteria. The dual meaning of Π results because d*P*/d*t* is scaled to have a maximum value of 1 in the absence of damage (*k*_0_ = λ = 0 in Equation 2a). The scaling assumes that *P* increases linearly with time in the absence of damage and also renders time into units of *P*. Although the true regulator of bacterial division is not known [15], [16], a model requiring the build-up of a product to a checkpoint is reasonable [11]. Various cellular (volume, mass and length) and biochemical attributes have been postulated to serve as checkpoints, but distinguishing between primary (causative) and secondary (downstream) regulators has been difficult. Regardless, the constancy of bacterial cell size shows that some accounting mechanism and a checkpoint must exist.

Upon dividing, the mother cell partitions her damage *D*_0_ to two daughters and
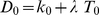
To allow for variation in the partitioning, let *a* and (1−*a*) be the proportion of *D*_0_ given to the daughters, which are subscripted 1 and 2 and 0≤*a*≤½. Thus, daughter 1 always receives less damage if *a*<½ and the damage given to the daughters is
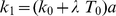
(3a)

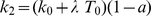
(3b)Because each daughter in turn becomes a mother, Equation 2b can be subscripted to describe the daughters or
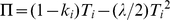
(4)

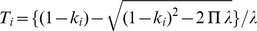
(5)by the quadratic formula and *i* = 1 or 2.

Thus, given *T*_0_ for a mother cell, *T_i_* of her two daughters can be determined. *k*_0_ in Equations 3a and 3b is obtained by rearranging Equation 2b as
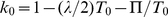
(6)The ability to predict *T*_1_ and *T*_2_ given *T*_0_ allows projecting forward in time the doubling time, and hence fitness, of every individual in a population. *T*_1_ and *T*_2_ serve as *T*_0_ for the next generation and Equations 3, 5 and 6 only need to be reiterated. Equations 3, 5 and 6 are hereafter referred to as the model.

## Results

### Equilibrium Conditions

To determine if a lineage of dividing cells converged to a determinable level of damage over successive generations, the model was examined for equilibria. Daughter cells reach an equilibrium when 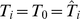 and 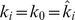. Substituting these conditions into Equations 3a and 3b, yields
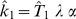
(7a)

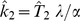
(7b)where α = *a*/(1−*a*). Substituting Equations 7a and 7b into Equation 4
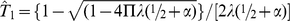
(8a)

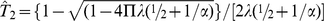
(8b)Equilibrium values 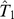 and 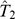 are possible if roots to the quadratic solutions of Equations 8a and 8b are real or
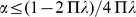
(9a)

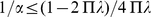
(9b)Thus, equilibria are possible, depending on the level of asymmetry and the product of the two parameters Π and λ. The linking of Π and λ into a single product or fundamental parameter facilitates the analysis of the model by reducing the effective number of parameters in the model from three to two. Because λ is the intrinsic damage rate and Π is the doubling or life time of a damage-free individual (Equation 2b), all damage in such a cell is acquired over its lifetime and Πλ represents its total or lifetime damage rate.

### Equilibrium with Symmetrical Transmission

The partitioning of damage from the mother cell to her daughter cells is symmetrical if *a* = ½, in which case the daughters are identical, *k*_1_ = *k*_2_, and *T*_1_ = *T*_2_. Letting *i* = 1 represent both daughters, the equilibrium conditions are provided by Equation 8a with α = 1 or
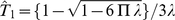
(10)The roots to Equation 10 are real if
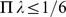
(11)


**Figure 1:**
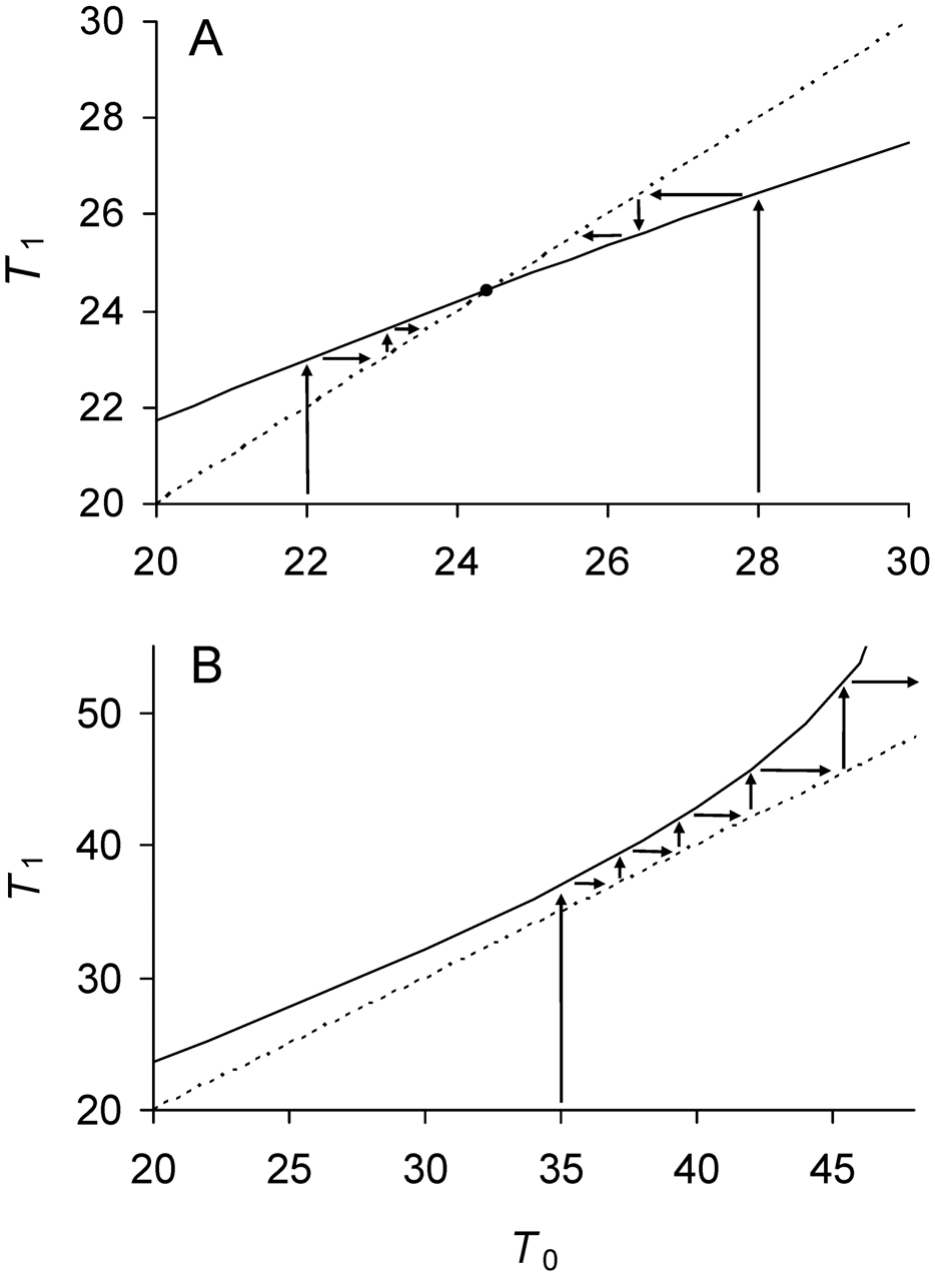
Phase plot of relationship between doubling times of daughter and mother cells when division is symmetrical. Daughter cell doubling time *T*_1_ predicted by Equations 3, 5, and 6 for a given mother doubling time *T*_0_ (——) with symmetry. One solid line is presented because with symmetry both daughter cells have the same doubling time. Time is measured in minutes. See Table 1 for complete definitions of model variables and parameters. (A) Low damage rates; *a* = ½, Π = 20, λ = 0.005, and Πλ = 0.10. Dynamics of bacterial lineages can be represented on the phase plane by mapping *T*_0_ to the solid line to derive *T*_1_. The next cell division of the lineage is obtained by allowing the daughters cells to become mother cells and divide, which is accomplished graphically by projecting *T*_1_ to the *T*_0_ axis. The projection is facilitated by mapping *T*_1_ to the identity line (----; *T*_1_ = *T*_0_). For example, if *T*_0_ = 22 min, the predicted *T*_1_ is 23 min. Projecting 23 min to identity line and then upwards to the solid line yields 23.6 min, which corresponds to the *T*_1_ of the next division. The successive divisions lead the lineage to the intersection of the solid and identity lines at 24.5 (•). The increase in doubling times corresponds to aging. The intersection denotes the stable equilibrium point 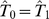 because a mother cell starting at *T*_0_ = 28 tracks backwards to the same point by a process of rejuvenation. The equilibrium is locally stable if the slope of the solid line at the intersection is less than one [34]. The equilibrium value of 24.5 is also predicted by Equation 10 by letting Π = 20 and λ = 0.005. This equilibrium is possible in the model because the lifetime damage rate of Πλ = 0.10 is less than the threshold of 1/6 (Equation 11). (B) High damage rates; *a* = ½, Π = 20, λ = 0.009, and Πλ = 0.18. Because Πλ>1/6, Equation 11 is not satisfied, the solid line does not cross the identity line, and 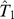 does not attain a real value. If cell division were projected graphically into future generations, *T*_1_ increases to infinity.

The equilibrium can be locally stable and the stability can be assessed graphically (Figure 1A). The stability results because the doubling times in a lineage descending from a mother cell with a doubling time less than 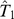 increase until equaling 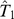. On the other hand, if the doubling time of a mother cell is greater than 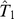, the doubling time of her lineage decreases to 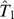.

If Πλ>1/6, the doubling time of a lineage never attains an equilibrium and it increases over generations until it is infinitely long (Figure 1B). When doubling time is infinitely long, a mother cell is alive but unable to divide because its damage content is too high and *P* cannot be built up to Π. At the threshold of Πλ = 1/6, *P* is built up to Π and, by Equation 10, 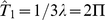, which corresponds to the equilibrium doubling time of the least fit symmetrical cell.

To obtain an estimate of the damage load as 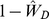 with symmetrical transmission, the equilibrium mean fitness 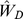 was estimated for a population of cells with doubling time of 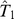 relative to a population with the highest fitness or the shortest possible doubling time of Π (Equation 2b). A population with doubling time of Π increases by definition by a factor of 2 during a time period Π. A population with a doubling time of 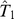 increases during the same period by a factor of 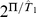. The mean relative fitness at equilibrium resulting from damage is therefore the ratio of 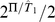, or
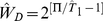
(12)

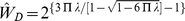
(13)by letting 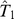 equal Equation 10.

A summary of the results, including new ones to follow, and the definitions of all parameters and variables for the model are presented in Table 1.

**Table 1:** Effects of parameter values on outcome of model.

Πλ	*a* = 0.5	0≤*a*<0.5
0	No damage. 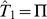 , 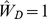 , and equilibrium mean fitness is highest possible.	Same as *a* = 0.5, except 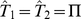 .
<1/6	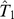 and 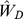 are real numbers and equilibrium values are achieved (Equation 10 and 13; Figure 1A).	If *a* is sufficiently large, 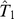 and 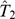 = real numbers and two separate equilibria are achieved ( 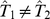 ; Figure 2A). Lineages are immortal (Equations 9a and 9b satisfied). If *a* is not sufficiently large, as below.
1/6	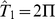 from Equation 10 and 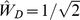 from Equation 12.	If *a* is not sufficiently large, 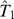 = real number and an equibrium is achieved (Equation 9a satisfied), but *T*_2_ increases to be infinitely long (Equation 9b not satisfied). Lineages can survive by reproduction, but they are mortal.
>1/6	Lineage cannot survive by division and *T*_1_ increases to be infinitely long (Equation 11 not satisfied; cf. Figure 1B).	If *a* is sufficiently small, same as above (Figure 2B). If *a* is not sufficiently small, same as below.
≥0.25	Same as above.	If *a* is not sufficiently small, lineage cannot survive by reproduction and both 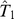 and 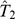 increase to be infinitely long.

Definitions of model variables and parameters: *DT* (doubling time); Π (shortest doubling time achieved by a bacterium free of damage); λ (damage rate); Πλ (multiplication product of the two parameters and the life time damage experienced by a damage-free bacterium); *a* (asymmetry coefficient); *T*_0_, *T*_1_, and *T*_2_ (doubling times of mother, daughter 1 and daughter 2 bacterium; **^∧^** (notation for equilibrium); 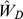 (equilibrium mean fitness of bacterial population experiencing damage). Results based on Equation 11 for *a* = 0.5 and Equations 9a and 9b for 0≤*a*<0.5.

### Transmission Rules with Asymmetry

**Figure 2:**
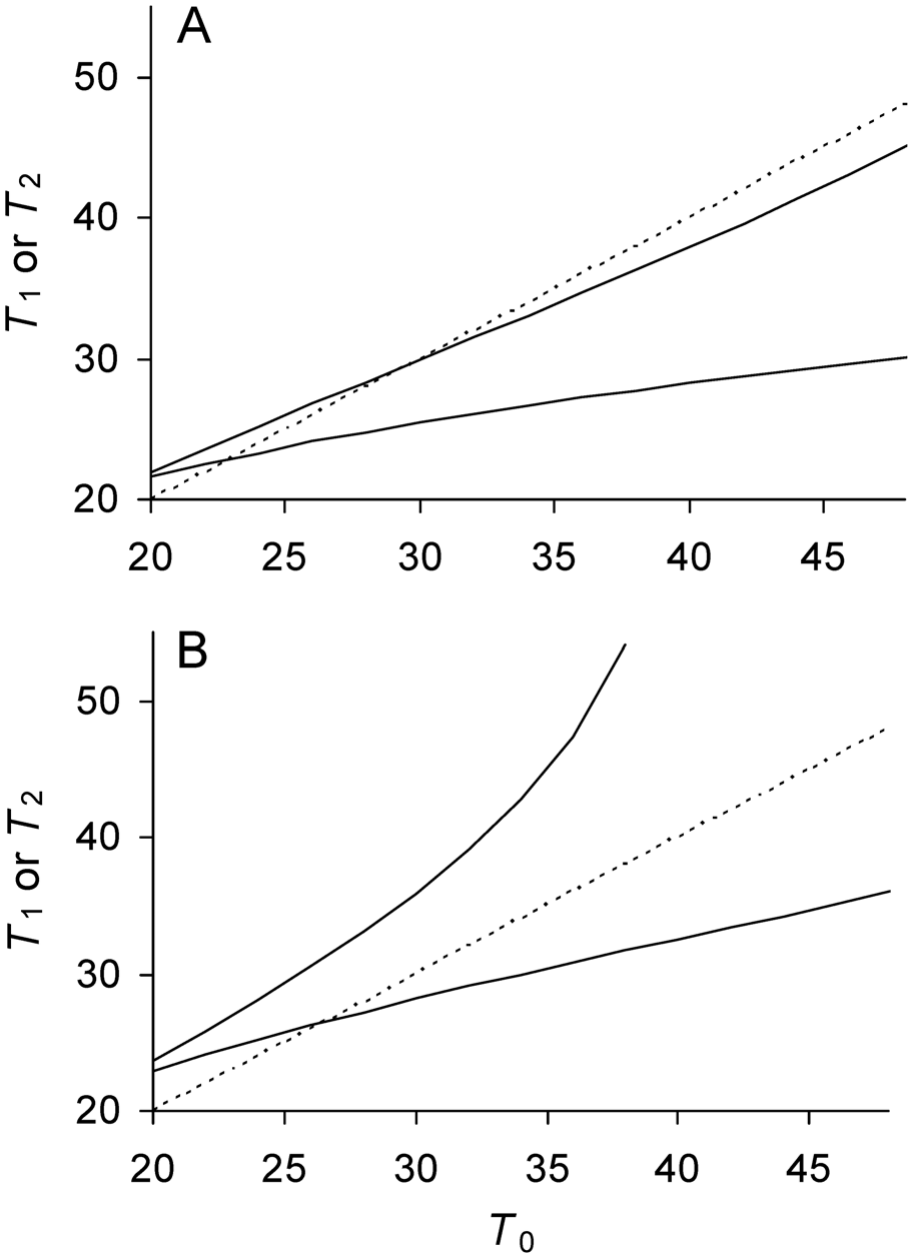
Relationship between doubling times of daughter and mother cells when division is asymmetrical. See Table 1 for definitions and Figure 1 for details and graphical projections. Identity line (----). With asymmetry, daughters 1 and 2 have different doubling times *T*_1_ and *T*_2_. Upper solid line is *T*_2_ (——); bottom solid line *T*_1_. (A) Low damage rates; *a* = 0.37, Π = 20, λ = 0.005, and Πλ = 0.10. Because Πλ<1/6 and *a* is sufficiently large (Table 1), Equations 9a and 9b are satisfied, both solid lines intercept the identity line. 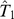 and 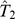 attain real values that are stable equilibria. If projected graphically as in Figure 1, *T*_1_ and *T*_2_ each converges to its equilibrium. (B) High lifetime damage rates; *a* = 0.37, Π = 20, λ = 0.0085, and Πλ = 0.17. Because Πλ>0.17 and *a* is sufficiently large, only Equation 9a is satisfied (Table 1). 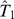 attains a real value and stable equilibrium, but 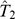 cannot and *T*_2_ would be projected to increase to infinity.

The evolution of the asymmetrical transmission of damage can be examined by letting 0≤*a*<½. Unlike *a* = ½ (Figure 1A), a separate equilibrium is now possible for each of the daughters (Equations 8a, 8b). Because *a*<½, daughter 1 gets less damage. Inspection of Equations 9a and 9b reveals that as Πλ increases from zero, 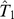 and 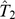 go through conditions in which both, one or none attain real equilibrium values (Table 1). If Πλ<1/6 and *a* is sufficiently large, Equations 9a and 9b are satisfied and both 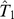 and 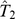 attain equilibria (Figure 2A). If *a* is not sufficiently large, only Equation 9a is satisfied and 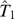 has an equilibrium while 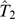 does not. The same outcome ensues if Πλ≥1/6 and *a* is sufficiently small (Figure 2B). Thus, the threshold of Πλ = 1/6 still plays an important role (Table 1).

**Figure 3:**
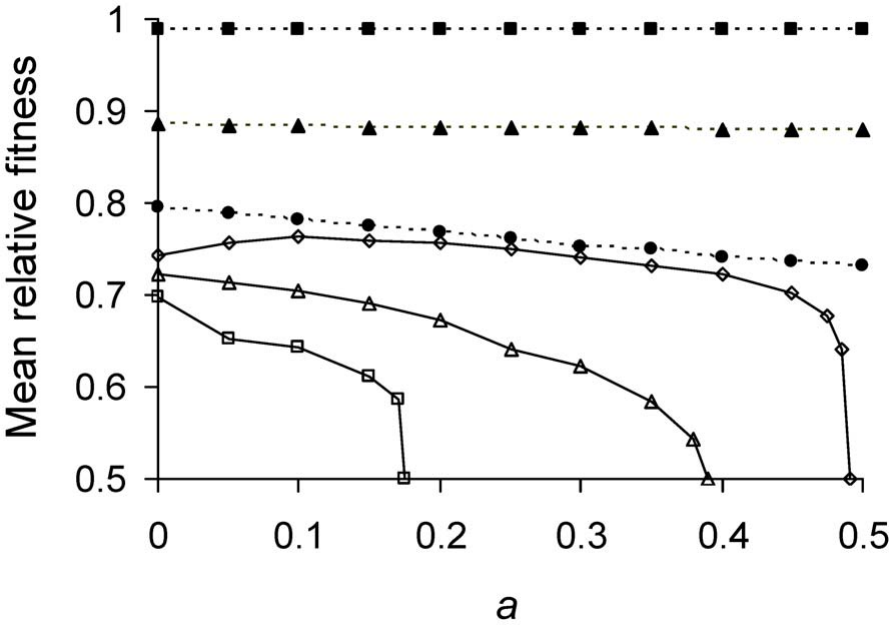
Mean relative fitness as function of asymmetry for varying lifetime damage rates. Πλ<1/6 (——); Πλ = 0.01 (▪), 0.1(▴), and 0.165 (•). Πλ>1/6 (----); Πλ = 0.17 (□), 0.21(Δ), and 0.24 (○). The highest fitness of 1 denotes a cell that divides into two daughters in Π minutes, the shortest possible doubling time manifested by a damage-free cell. The lowest fitness of 0.5 corresponds to a severely damaged cell that is alive but no longer able to divide. Thus, after Π minutes, former cell contributed two counts to the population while the latter contributed only one. Mean relative fitness 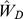 was obtained by simulating a population of cells under selection until a fitness equilibrium. The simulation tracked the doubling times of 1000 individual cells by applying Equations 3, 5, and 6. Selection was allowed to operate by having cells with shorter doubling times divide sooner. The population was randomly culled back to a size of 1000 immediately after the division of any cell. *T*_1_ and *T*_2_ values for individual bacterium were obtained from the simulation, converted to relative fitness (Equation 12), and averaged to obtain 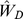, which remains a function of Πλ because the use of Equations 6 and 12 combine Π and λ into a product. The random culling renders death a stochastic process in the estimation of 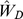. Simulations were also performed with a smaller population size of 200 individuals and no differences were obtained, which indicates that the outcome was due primarily to deterministic and not stochastic factors.

If 0≤*a*<½, unlike when *a* = ½, knowing the values 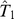 and 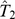 does not allow an estimate of 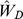. 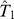 and 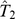 represent extreme values to which the doubling time of daughters converge as they replicate, e.g., as it would be illustrated if a graphical projection (see Figure 1) were applied to Figure 2. As new daughter 1 and 2 cells are generated, lineages descending from each type converge to 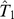 and 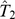, respectively, if the equilibria exist. If a daughter 1 is at the equilibrium 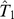, it still generates daughter 2 cells, which create new lineages that now converge onto 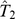. The presence of different lineages generates a population with mixed doubling times. The distribution of doubling times in the population is in turn shaped by natural selection and 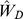 can be estimated only after the distribution reaches a selection-damage equilibrium. In contrast, if *a* = ½, 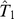 offers a direct estimate of 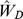 (Equation 10) because a population with mixed doubling times is not possible at equilibrium. Once a lineage converges to 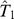 all descending daughters have a doubling time of 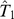.


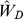
 was therefore estimated by using the model to simulate a population of cells under selection until a fitness equilibrium was reached. Selection was imposed by allowing cells with shortest doubling times to divide before other cells (Figure 3; Table 1). Πλ behaves again as a single parameter because the model uses Equation 5, which when combined with Equation 12 collapses Π and λ into a product. For values of 0<Πλ<1/6, 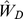 is highest with *a* = 0 and maximum asymmetry is favored. However, the advantage shrinks as Πλ decreases to zero. The difference between 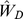 for *a* = ½ and *a* = 0 decreased from 8.6%, 0.57% to 0.0025% when Πλ goes from 0.165, 0.1 to 0.01. For Πλ>1/6, asymmetry is favored more strongly but relationship between 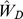 and Πλ is non-monotonic. When Πλ = 0.17, 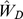 is maximized at *a* = 0.1 and an intermediate asymmetry is favored. Such non-monotonicity is also present, but less apparent, for other values of Πλ, including when Πλ<1/6.

Inspection of the relationship between 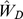 and *a* for extreme values reveals why it is not monotonic for Πλ>1/6. Simulations showed that for Πλ>0.25 populations were unable to grow, regardless of *a*. When Πλ>0.25 and *a* = 0, Equation 9a is satisfied and daughter 1 achieves its equilibrium of 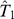. However, daughter 2 from a cell at 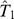 receives too much damage and is unable to divide. Thus, the mother cell just replaces herself with daughter 1 and there is no net reproduction. A similar effect explains the left side of the hump when Πλ = 0.17 (Figure 3). However, in this case 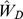 declines not because daughter 2 is unable to divide, but because the grand-daughter 2 is unable (analysis not presented). The right side of the hump for Πλ = 0.17 results because with low asymmetry Equation 9a is not satisfied and daughter 1 is now unable to divide. The combination of all these effects explains also many of the inflections seen in Figure 3.

### Estimating Model Parameters

**Figure 4:**
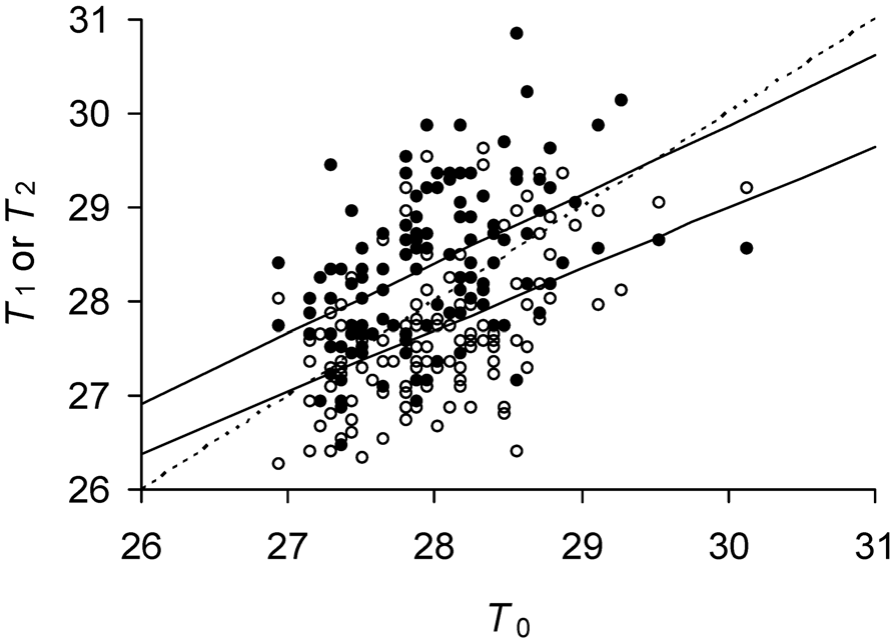
Predicted and observed doubling times of *E. coli* daughter and mother cells. Time is measured in minutes. Observed *T*_1_ (•) and *T*_2_ (○) as a function of observed *T*_0_. Predicted *T*_1_ (——, lower) and predicted *T*_2_, (——, upper) for a given *T*_0_. Observed doubling times were obtained from Stewart *et al* (*13*) by transforming reported growth rates for mother, daughter 1 and 2 cells into doubling times. Only the eighth and last doubling of the reported data was used because it provided the largest sample size of 2^8^ or 256 cells. Sample size n = 128 or the number of mothers for each plot. Predicted doubling times obtained from model with estimated values of Π, λ, and *a* (see Estimating Model Parameters). Intercepts between the identity line (----) and (——) line correspond to stable equilibrium points 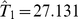 and 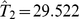 min (cf. Figure 2A).

Recent *E. coli* data [14] reporting growth rates of mother and daughter cells allow determining where in the parameter space of the model a biological organism resides. By converting the reported growth rates to doubling times, observed values of *T*_0_, *T*_1_ and *T*_2_ were obtained for the bacterium (Figure 4). Each observed *T*_0_ was then inputted into the model over the parameter space to derive expected *T*_1_ and *T*_2_ values. The parameters of the model were determined as those that minimized the difference between the observed and expected *T*_1_ and *T*_2_ by maximum likelihood via a conjugate gradient method implemented in the software package HyPhy [17]. The parameter *a* was estimated to have a mean value of 0.4836 and a 95% Bayesian Confidence Interval (BCI) of [0.4716–0.4905]. Estimated mean value of Π was 18.95 min (95% BCI [16.61–21.71]) and of λ was 0.007737 min^−1^ (95% BCI [0.005347–0.009717]). Applying these mean values of Π, λ, and *a* to Equations 8a and 8b estimated of 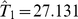 and 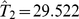 min, which show that the doubling times of the two daughter lineages attained separate equilibria. The presence of these two equilibria in *E. coli* was also revealed by a phase plot overlaying the observed and expected values (Figure 4).

**Figure 5:**
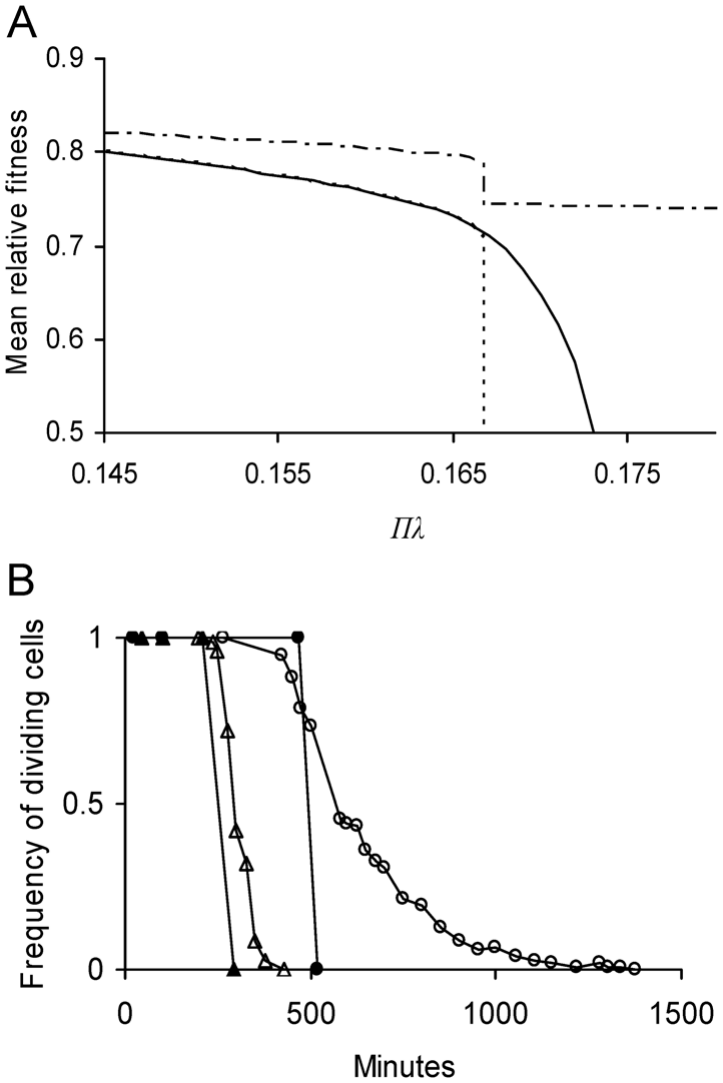
Predicted effect of lifetime damage rate on fitness and persistence of *E. coli* cells. (A) Equilibrium mean fitness 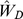 as a function of lifetime damage rate Πλ and asymmetry coefficient *a*. See Figure 3 for methods used in determining 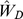. A value of 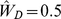 denotes again no cell division. Because a symmetrical cell is unable to divide if Πλ>1/6 (Equation 11), its equilibrium mean fitness drops to 0.5 as Πλ increases to the threshold (----). Because asymmetry allows bacteria to tolerate a higher damage rate (Table 1), *E. coli* with its estimated asymmetry of *a* = 0.4836 (see Estimating Model Parameters), it is able to divide up to Πλ = 0.173 (——). For the estimated values *a* = 0.4836 and Πλ = 0.1467, 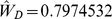. For a symmetrical cell with equivalent lifetime damage (*a* = 0.5 and Πλ = 0.1467), 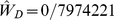. Thus, the evolved asymmetry estimated for *E. coli* only elevates 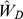 by a difference of 3×10^−5^, which is too small to resolve in the figure. However, the advantage of evolving an extreme asymmetry is great because a cell with *a* = 0 is able to divide over the entire range of presented Πλ values (— - —). (B) Frequency of dividing cells as a function of time for damage-free cells suddenly challenged with a high damage rate. Results compare a symmetrical cell and one with the asymmetry estimated for *E. coli*. Frequency of dividing cells was determined by iterating the model over successive generations in an initially damage-free population. Cells were considered non-dividing when their doubling times became infinitely long. If Πλ = 0.175, symmetrical cells were unable to divide after 516 min (—•—) while an *E. coli* equivalent was able up1378 min (—○—), a 63% advantage. If Πλ were elevated to 0.185, symmetrical cells stopped after 296 min (—▴—) and an *E. coli* equivalent after 426 min (—▵—), a 31% advantage.

Applying the estimates of Πλ = (18.95)(0.007737) = 0.1467 and *a* = 0.4836 to the model also showed that 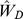 was higher, though only by a small amount, for these *E. coli* relative to a symmetrical (*a* = ½) bacterium with the same value of Πλ (Figure 5A). However, the advantage became greater if Πλ were increased. While bacteria with *a* = ½ could not reproduce once Πλ>1/6 (Equation 11), these *E. coli* were able up to Πλ = 0.173 (Figure 5A). If Πλ>0.173, neither bacteria could reproduce, but damage accumulated and shut down division more slowly in cells with *a* = 0.4836 than *a* = ½ (Figure 5B).

## Discussion

Just as deleterious mutations and selection create at equilibrium a mutational load, non-heritable damage and selection generate a damage load. Both type of loads contribute to the phenotypic load [18]. Analysis of a model for damage load in organisms dividing by fission revealed that a single fundamental parameter equal to Πλ determines equilibrium mean fitness 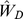, where the damage load is 1−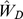 (see Table 1 for summary). Πλ is the product of two separate parameters in the model, λ the intrinsic damage rate and Π the doubling or life time of a damage-free individual. Thus, Πλ represents the total or lifetime damage rate of damage-free cell.

A damage load selects for mother cells that partition damage asymmetrically between daughters. If division were symmetrical, the daughters are identical and the equilibrium doubling time 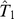 of descending lineages attains a real value when lifetime damage rate is less than 1/6 (Figure 1A). If the rate is greater 1/6, 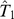 becomes infinitely long because the mother cells acquire too much damage and are unable to build cellular products to the amount needed for fission. The cell is alive but the lineage dies because doubling time becomes infinitely long (Figure 1B). Asymmetry allows cells to survive by division up to a rate less than 0.25 (see Transmission Rules; Figure 2; Table 1). Moreover, within the range of 0≤Πλ<0.25, equilibrium mean fitness is generally maximized as asymmetry decreases to the extreme when one daughter receives all of the damage harbored by a mother cell. Such extreme asymmetry is represented in the model with an asymmetry coefficient with a value of *a* = 0. However, for some intermediate lifetime damage rates an optimal and intermediate value of asymmetry is favored (Figure 3; Πλ = 0.17).

The evolution of asymmetry due to a damage load is comparable to the evolution of sex from a mutational load. The evolution of sex requires that the distribution of deleterious mutations is underdispersed in a population, i.e., that the variance is less than the mean [2]. Because sex shuffles genetic variation, its net effect is to redistribute mutations by a Poisson process, in which case the variance converges to the mean. If the variance and mean are equal, sex is not advantageous because the variance cannot be changed. If sexual reproduction were to overdisperse deleterious mutations an advantage can result, but that is prevented by the rules of genetic transmission. Asymmetry likewise increases variance in a population, but selection for asymmetry is much stronger because transmitting all or none of the damage to the daughters overdisperses the variance.

Asymmetrical transmission impacts the life history of a lineage by creating the two types of daughters. While daughter 1 is rejuvenated at birth, daughter 2 is loaded with damage. The deterioration of daughter 2, her daughter 2 in turn, and so forth constitutes currently one of the main hypothesis for the evolution of aging or senescence in microbes [11]–[14]. Fission in microbes results in the creation of a new and old pole. Because the new pole harbors less damage, it tags daughter 1. However, the long term consequences of asymmetry are debated. With symmetry and low damage, single-celled organisms are immortal (Figure 1A). Do high damage and asymmetry make them mortal [14], [19]–[22]? The model shows that it depends on the level of asymmetry and the rate of lifetime damage. If lifetime damage rate is less than 1/6 and asymmetry is sufficiently large, both the equilibrium doubling times 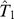 and 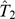 of daughters 1 and 2 attain real values and the microbe is immortal (Figure 2A). If the asymmetry is not sufficiently large, 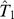 has a real equilibrium value, but 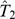 becomes infinitely long and the daughter 2 lineage is mortal. The same outcome ensues if lifetime damage rates are greater than or equal to 1/6 and asymmetry is sufficiently small (Figure 2B). The mortality of daughter 2 renders all lineages in microbe mortal because all new poles eventually become old and reside in a daughter 2. Thus, although asymmetry matters, a lifetime damage rate of 1/6 is a key threshold. If the rate is less than 1/6, immortality is possible. If the rate is greater than or equal to 1/6, a microbe is mortal.

A recent study recording the division of *E. coli* cells over generations [14] provided an estimate of parameters for lifetime damage rate and asymmetry (see Estimating Model Parameters). The estimates placed *E. coli* in an area of the parameter space where the bacterium was immortal; both 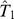 and 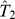 attained real equilibrium values (Figure 4). However, the estimate of the asymmetry coefficient *a* at a value of 0.4836 was at first surprising. Given that fitness is generally maximized with extreme asymmetry (*a* = 0; Figure 3), a lower *a* could have been expected. What is the level of advantage provided by such a small degree of asymmetry? Could the level of asymmetry just be noise rather than an adaptation [22], [23]? If asymmetry is adaptive, why has it not evolved to be much higher? Resolution of these issues requires more information, but the current model can be used to provide guidance at this point.

On the basis of the parameter values estimated, the model predicts that the equilibrium mean fitness for an asymmetrical *E. coli* with *a* = 0.4836 is higher by a difference of 3×10^−5^ when compared to that of a hypothetical and symmetrical *E. coli* with *a* = ½ (Figure 5A). Although small, such a difference is more than sufficient for evolving asymmetry in large microbial populations. However, the difference may be on the low end of the range experienced by *E. coli* because the model was based on parameter values estimated in a benign laboratory environment. The parameter lifetime damage rate was estimated to be 0.1467, but it could be much higher for *E. coli* in the wild. If the rate were increased by 14% to the threshold of 1/6 (Equation 11), the advantage of a small degree of asymmetry is magnified. At this new rate, a symmetrical *E. coli* can no longer persist by reproduction and its doubling time becomes infinitely long (Figure 1B). With an asymmetry of just 0.4836, asymmetrical *E. coli* can persist up to rates as high as 0.173 (Figure 5A). Moreover, the advantage continues to increase if rates were further elevated. If they were greater than 0.173, *E. coli* with both *a* = 0.4836 and symmetry cannot persist, but damage accumulates and cell division shuts down more slowly in the asymmetrical bacterium. For example, if the rate were 0.175, the frequency of dividing cells drops to 0% in slightly over 500 min with symmetry, but only after more than 1300 min with *a* = 0.4836 (Figure 5B). Retaining a few dividing cells for several extra hundred minutes could be invaluable to an organism capable of rapid growth. Thus, the fitness advantage of a small degree of asymmetry, such as *a* = 0.4836, could be high.

However, why has asymmetry level in *E. coli* not evolved to be higher than 0.4836? An obvious answer is there may be a cost. Although the model assumes no costs, it reveals the fitness constraints. Assuming that *E. coli* experiences a higher lifetime damage rate of 0.17, inspection of Figure 3 shows that the equilibrium mean fitness has the greatest curvature when asymmetry equals 0.475. Equilibrium mean fitness equals 0.68 when asymmetry is 0.475 and it increases only by 3% to 0.70 when asymmetry is 0.450. If in the simplest scenario the cost of reducing *a* from ½ to 0.475 equals from 0.475 to 0.450, fitness gain for the second reduction could be too small to override the costs. The fitness gain for the first reduction is large because symmetrical bacteria cannot reproduce when the lifetime damage rate is 0.17 (Figure 3; Table 1). Unless the cost is extremely small or the second reduction is less costly, asymmetry should evolve to reside where the curvature is greatest [24]. The level of asymmetry can be shifted by different cost functions, but the curvature constrains its evolution to the neighborhood of 0.475. Estimating the costs will be needed for a full resolution, but the low asymmetry estimated for *E. coli* may well be anticipated by the model.

A higher lifetime damage rate may be reasonable for *E. coli* and other microbes. Defenses and weapons by microbial competitors and hosts routinely employ mechanisms that inflict non-genetic damage often through oxidation [25]–[29]. Microbes face damage even in apparently benign environments. *E. coli* grown under standard laboratory conditions do not experience much oxidative damage. However, 48 hr after reaching stasis, oxidative damage to proteins increases six fold [30]. The damaged population can be separated into two fractions by centrifugation. One fraction, which accounts for 40% of the cells, contains bacteria capable of dividing and forming colonies on agar plates. The bacteria in the second fraction are not, although they remain intact and metabolically active. Most importantly, almost 90% of the detectable oxidative damage is in the second fraction, which demonstrates well outcomes comparable to Figure 2A and Figure 5B.

Because a damage load is created by non-heritable variation, it has characteristics that are attributable to the soma. The asymmetrical transmission of non-heritable damage in microbes, and the subsequent division of labor [31], has led to suggestions that these organisms have the equivalent of soma and germline [21],[32]. From this perspective, the evolution of germline, soma, and senescence in metazoans [32] is just the extension of microbial asymmetry and the damage load could be called the somatic load. Although the present model was formulated for microbes, it could be generalized to include metazoans. It may also be useful for describing a population of cells within a metazoan. Do stem cells partition damage asymmetrically?

Many aspects of the present model are not novel. Previous models have shown that the asymmetrical transmission of damage can be favored during binary fission by both directional and stabilizing selection [8]–[10]. Erjavec *et al.* [11] demonstrated qualitatively with simulations a threshold for cells dividing symmetrically. However, the present model offers some new perspectives. First, the derivation of a damage load allows a comparison to a mutational load. In metazoans with large genomes, the mutational load [33] could be as large as some of the damage load estimates (Figure 3). On the other hand, because the mutational load is smaller in microbes [33], the damage load could be a stronger evolutionary force. Second, the present model shows that the two parameters Π and λ combine to form a single fundamental parameter as the product Πλ or the lifetime damage rate. Moreover, the model was also able to predict key thresholds for Πλ at 1/6 and 0.25 without any empirical/data calibration. The threshold of 1/6 delineates the boundary for when cell dividing by fission is mortal or immortal. This outcome stands in contrast to the absence of any theoretical framework for whether the genomic mutation rate *U*, a key parameter for determining the mutational load (Equation 1) has an upper limit, despite the fact that metazoans and RNA viruses have independently evolved maximum rates of 1 (reference [33]). Third, a fit of the model to experimental data from *E. coli* provided estimates for all of the key parameters in the model. The parameter values showed that *E. coli* was immortal under the conditions examined. The determination of where a real organism resides in parameter space offers a powerful predictive tool for studying evolution.

## References

[1] Haldane JBS (1937). The Effect of Variation of Fitness.. The American Naturalist.

[2] Kondrashov AS (1993). Classification of Hypotheses on the Advantage of Amphimixis.. Journal of Heredity.

[3] Muller HJ (1950). Our Load of Mutations.. American Journal of Human Genetics.

[4] Crow JF (1958). Some possibilities for measuring -selection intensities in man.. Human Biol.

[5] Kimura M, Maruyama T (1966). Mutational Load with Epistatic Gene Interactions in Fitness.. Genetics.

[6] Ohta T (1973). Slightly Deleterious Mutant Substitutions in Evolution.. Nature.

[7] Charlesworth B, Charlesworth D (1998). Some evolutionary consequences of deleterious mutations.. Genetica.

[8] Ackermann M, Chao L, Bergstrom CT, Doebeli M (2007). On the evolutionary origin of aging.. Aging Cell.

[9] Evans SN, Steinsaltz D (2007). Damage segregation at fissioning may increase growth rates: A superprocess model.. Theoretical Population Biology.

[10] Watve M, Parab S, Jogdand P, Keni S (2006). Aging may be a conditional strategic choice and not an inevitable outcome for bacteria.. Proc Natl Acad Sci U S A.

[11] Erjavec N, Cvijovic M, Klipp E, Nystrom T (2008). Selective benefits of damage partitioning in unicellular systems and its effects on aging.. Proc Natl Acad Sci U S A.

[12] Ackermann M, Stearns SC, Jenal U (2003). Senescence in a bacterium with asymmetric division.. Science.

[13] Lindner AB, Madden R, Demarez A, Stewart EJ, Taddei F (2008). Asymmetric segregation of protein aggregates is associated with cellular aging and rejuvenation.. Proc Natl Acad Sci U S A.

[14] Stewart EJ, Madden R, Paul G, Taddei F (2005). Aging and death in an organism that reproduces by morphologically symmetric division.. Plos Biology.

[15] Boye E, Nordstrom K (2003). Coupling the cell cycle to cell growth - A look at the parameters that regulate cell-cycle events.. Embo Reports.

[16] Vinella D, Dari R (1995). Overview of Controls in the Escherichia-Coli Cell-Cycle.. Bioessays.

[17] Pond SLK, Frost SDW, Muse SV (2005). HyPhy: hypothesis testing using phylogenies.. Bioinformatics.

[18] O'Donald P (1968). Measuring the intensity of natural selection.. Nature.

[19] Kirkwood TB (2005). Asymmetry and the origins of ageing.. Mech Ageing Dev.

[20] Ferber D (2005). Microbiology. Immortality dies as bacteria show their age.. Science.

[21] Nystrom T (2002). Aging in bacteria.. Curr Opin Microbiol.

[22] Woldringh CL (2005). Is Escherichia coli getting old?. Bioessays.

[23] Stewart E, Taddei F (2005). Aging in Esherichia coli: signals in the noise.. Bioessays.

[24] Charnov EL (1976). Optimal Foraging, Marginal Value Theorem.. Theoretical Population Biology.

[25] Gross L (2007). Paradox Resolved? The Strange Case of the Radiation-Resistant Bacteria.. PLoS Biology.

[26] Lambeth JD (2004). NOX enzymes and the biology of reactive oxygen.. Nat Rev Immunol.

[27] Park B, Nizet V, Liu GY (2008). Role of Staphylococcus aureus catalase in niche competition against Streptococcus pneumoniae.. J Bacteriol.

[28] Dukan S, Farewell A, Ballesteros M, Taddei F, Radman M (2000). Protein oxidation in response to increased transcriptional or translational errors.. Proc Natl Acad Sci U S A.

[29] Doke N, Miura Y, Sanchez LM, Park HJ, Noritake T (1996). The oxidative burst protects plants against pathogen attack: mechanism and role as an emergency signal for plant bio-defence–a review.. Gene.

[30] Desnues B, Cuny C, Gregori G, Dukan S, Aguilaniu H (2003). Differential oxidative damage and expression of stress defence regulons in culturable and non-culturable Escherichia coli cells.. EMBO Rep.

[31] Kirkwood TB (2005). Understanding the odd science of aging.. Cell.

[32] Turke PW (2008). Williams's theory of the evolution of senescence: Still useful at fifty.. Quarterly Review of Biology.

[33] Drake JW, Charlesworth B, Charlesworth D, Crow JF (1998). Rates of spontaneous mutation.. Genetics.

[34] May RM (1976). Simple Mathematical-Models with Very Complicated Dynamics.. Nature.

